# Lithography based resonant marker design for MRI catheter visualization

**DOI:** 10.1186/1532-429X-18-S1-P208

**Published:** 2016-01-27

**Authors:** Engin Baysoy, Dursun K Yildirim, Cagla Ozsoy, Adrienne C Washburn, Anthony Z Faranesh, Senol Mutlu, Robert J Lederman, Ozgur Kocaturk

**Affiliations:** 1 grid.11220.300000000122539056Institute of Biomedical Engineering, Bogazici University, Istanbul, Turkey; 2 grid.279885.90000000122934638Cardiovascular and Pulmonary Branch, Division of Intramural Research, National Heart Lung and Blood Institute, Bethesda, MD USA

## Background

The unavailability of safe, conspicuous devices remains the chief barrier to wider clinical adoption of interventional cardiovascular magnetic resonance imaging. Active catheter receiver coils are conspicuous but their metallic transmission lines risk heating and their manufacture requires excessive device size. We have designed a clinical grade 5 Fr radio frequency (RF) resonator marker embedded directly into the catheter shaft using novel lithography techniques. We developed special manufacturing techniques to impart these circuits onto non-planar catheter surfaces. This approach eliminates (1) the bulky circuit components such as wire-wound coils and rigid capacitors as well as (2) long conductive cables, required for receiver coil-based visualization, that are subject to heating.

## Methods

**Figure 1:**
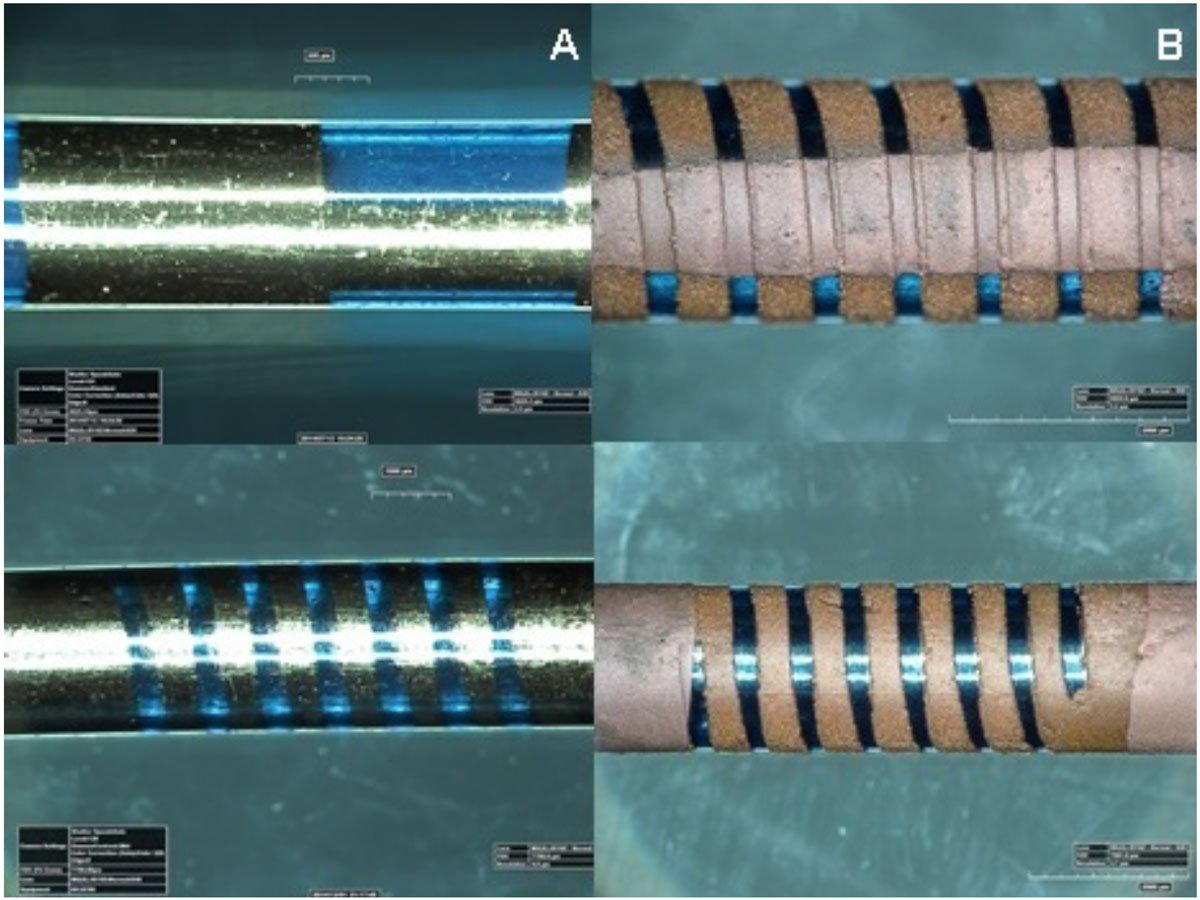
**Photograph of the novel resonant marker prototype first coated with (A) a seed layer (Chromium and Gold) in a capacitor form (top) and a solenoid form (bottom) and (B) Copper Electroplated form (front view (top) and back view (bottom))**.

The resonant distal tip marker was designed as a conventional LC tank resonant circuit (1-3) by incorporating a solenoid and a capacitor formed over non-planar 5 Fr catheter shaft surface using lithography techniques. The solenoid coil and the appropriate capacitor dimensions were determined based on the simulation results (Comsol Inc.). A planar physical vapor deposition (PVD) system (NVTS, Nanovak, Turkey) was modified to perform thin film coating on catheter surfaces. The thin film coating masks of each layer were constructed using polyamide tubing for the sputtering process. Chromium (11 nm thickness) and Gold (218 nm thickness) metallic conductors were coated as seed layers to form the solenoid coil and matching capacitor over the catheter shaft (Figure 1A). The seed layers were then electroplated with copper (15 µm thickness) to lower the resistance (Figure 1B). Parylene-C dielectric was applied as insulation layers. The resonant marker sensitivity (S11, reflection coefficient) was measured with a sniffer coil and a network analyzer (4395A, Agilent). The visibility performance of the resonant markers were tested in a water filled phantom and MR heating of the resonant markers were compared with a typical solenoid and transmission-line "active" MRI 5 Fr catheter in ASTM 2182 gel phantom using GRE sequence. (TE/TR =1.8/8.2 ms, flip = 5°).

## Results

**Figure 2:**
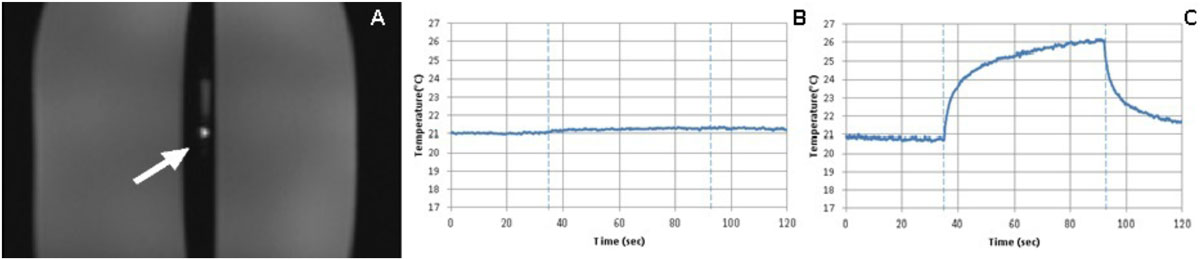
**(A) MR imaging of resonant marker (white arrow), (B) RF induced heating test of lithography based resonant marker (ΔT=0.35 °C), (C) active catheter marker (ΔT=5.34 °C) performed in water filled phantom**. (Dash lines indicate initiation and termination of MRI scanning)

The resonant frequency of the resonant marker was tuned to 63.66 MHz and the S11 measurement was -0.46 dB in loaded conditions, indicating that most transmitted power was reflected back by the resonator. The resonant distal tip marker prototypes provided satisfactory device tip signal (Figure 2a). The novel resonant marker design catheter exhibited no detectable heating (Figure 2b) while a comparable-profile active catheter heated 5.3 ^o^C under these test conditions (Figure 2c). in vivo data will be presented.

## Conclusions

We have successfully developed a lithography based technique to manufacture low profile resonant visibility markers directly onto catheter shafts. The final design provides satisfactory distal tip visibility under MRI and does not cause detectable RF induced heating compared with a comparable-profile active device under MRI.

